# The Association Between Neighborhood Gunshot Frequency and the Development of Preterm Birth

**DOI:** 10.1177/26884844251366375

**Published:** 2025-08-14

**Authors:** Brian W. James, Rachel Fisher, Chishu Yin, Brittany L. Kmush, Robert Silverman, Dimitrios Mastrogiannis

**Affiliations:** ^1^ Department of OBGYN, SUNY Upstate, Syracuse, New York, USA.; ^2^ Norton College of Medicine, SUNY Upstate, Syracuse, New York, USA.; ^3^ Department of Mathematics, Syracuse University, Syracuse, New York, USA.; ^4^ Department of Public Health, Syracuse University, Syracuse, New York, USA.; ^5^ Department of Maternal-Fetal Medicine, SUNY Upstate, Syracuse, New York, USA.

**Keywords:** gunshots, preterm birth, risk factors

## Abstract

**Objective:**

To determine the association between stress, as objectively measured by frequency of neighborhood gunshots and preterm birth (PTB).

**Study Design:**

A retrospective chart review of 1675 individual births was analyzed of pregnant women who lived in the City of Syracuse, New York, United States. The frequency of gunshots was measured in the acute phase (within 1 week of delivery) and the chronic phase (sum total of all gunshots in the previous 2 years). Primary outcome includes PTB prior to 37 weeks of gestation. Secondary analysis includes delivery prior to 34, 32, and 28 weeks of gestation.

**Result:**

Gunshots were significantly different between the three districts of Syracuse, which matched with differences in socioeconomic and comorbid conditions. The different districts also experienced differences in frequency of PTB (highest 18%, medium 13%, lowest 12%, *p* = 0.018). However, those with versus without PTB at any gestational age did not differ in the frequency of gunshots in acute phase or chronic phase.

**Conclusion:**

The use of acute-phase and chronic-phase gunshots as a method to simulate stress levels is not associated with the development of PTB.

## Introduction

Preterm birth (PTB) has been shown to be associated with increased morbidity and mortality in newborns.^1–4^ Issues of prematurity include neonatal respiratory distress syndrome, necrotizing enterocolitis, retinopathy of prematurity, and many other neurological, behavioral, and developmental challenges.^5^ Risk factors associated with spontaneous PTB include history of PTB,^6–8^ non-White race,^5,9^ lower socioeconomic standing,^6^ lower maternal weight,^10^ and tobacco use.^11,12^ Measurements of cervical length^13,14^ and fetal fibronectin^15^ are tools for predicting impending preterm labor but have significant limitations. Though risk models have been developed, it is still very difficult to predict who is most at risk of developing PTB before it occurs.^16,17^

Previous research has suggested that allostatic load (stressful environments and lifestyles) may lead to numerous adverse health outcomes, including cardiovascular diseases, cancer, and overall morbidity and mortality among the general population.^18–20^ Differences in individuals and local neighborhood environments can result in a similar variety of negative pregnancy outcomes.^21^ It has also been proposed that increasing stress levels can lead to increased incidence of pregnancy-induced hypertension,^22^ small for gestational age,^22^ and PTB.^23–25^ However, a majority of these studies use self-reported stress levels *via* questionnaires, which can be inaccurate and misleading.^26^ Prior studies have utilized neighborhood-specific gunshot violence data as objective and verified proxies for stress levels and many other factors that can influence overall well-being,^27,28^ including for pregnant women.^29^ The surgeon general recently published an advisory regarding the dangers that gunshot violence has to overall public health.^30^

The goal of this study is to determine whether stressful living situations, as objectively measured by local gunshot frequency, also increase pregnant women’s likelihood of being diagnosed with PTB. We postulate that there is an increased proportion of women who develop PTB living in areas with higher frequencies of gunshot per capita.

## Methods

**FIG. 1.**
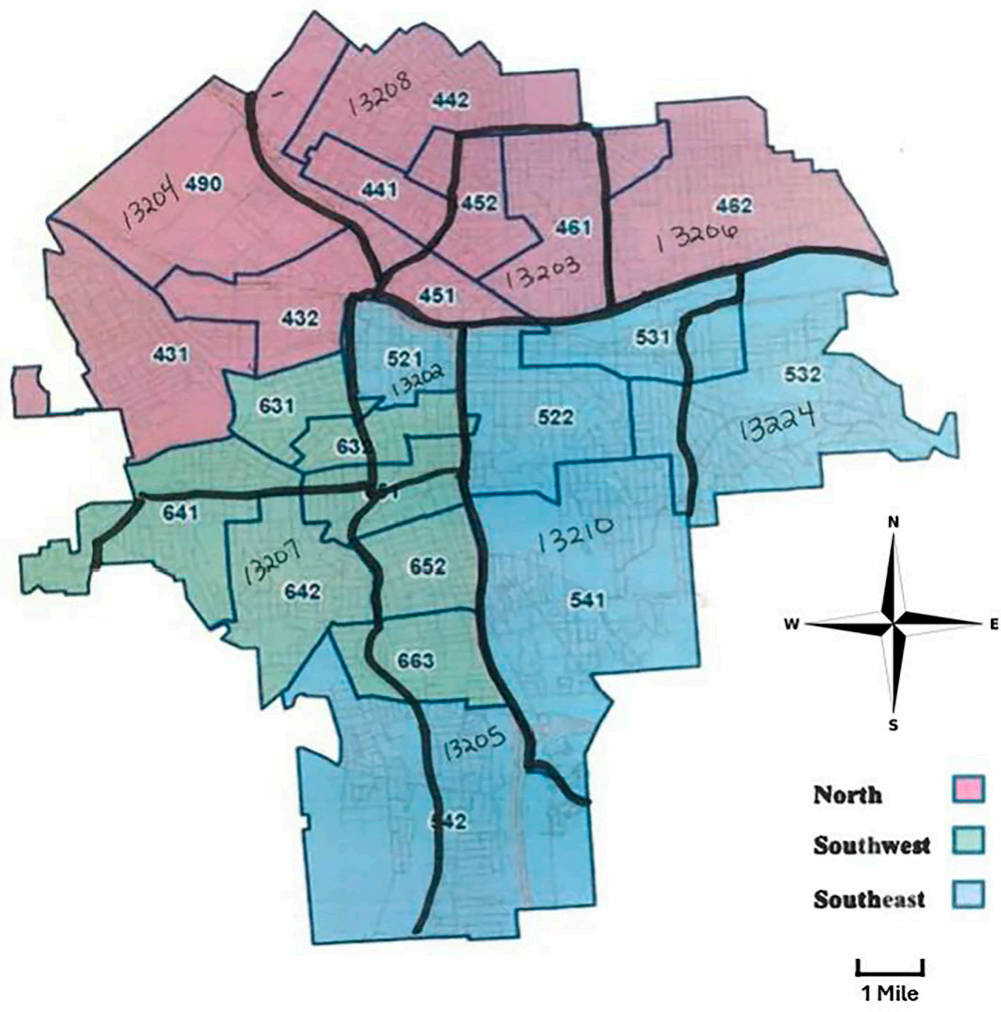
Zip-district zones across the city of Syracuse, 2020.

The study was approved by the Crouse Hospital’s Institutional Review Board. Informed consent requirement was waived given the retrospective nature of the data and the lack of participant identifiers. This was a retrospective cohort study of all women aged 18 years or older who live in the City of Syracuse, New York, United States, and delivered a viable infant at Crouse Hospital Labor and Delivery between August 2020 and August 2022. This hospital is a tertiary referral center and hosts the region’s perinatal center. Data were obtained *via* chart review of electronic medical records and included basic demographic data and zip code. These data were requested and received from Crouse Hospital’s Senior Quality Improvement Coordinator, who supplied this information *via* a secure password-protected e-mail with a set expiration date. The specific living area was subdivided based on the Onondaga County Board of Elections mapping of Syracuse into three distinct districts: North, Southwest, and Southeast,^31^ sometimes intersecting through a given national zip code (Fig. 1).

All participants who met the inclusion criteria were identified *via* a medical record system review of charts, and medical record numbers were collected. These medical record numbers were stored in a secure file drive on a password-protected computer inside a locked office space. Once the chart abstraction was completed, each participant was assigned an arbitrary participant number, and their medical record number was deleted.

The primary outcome of PTB, as defined by birth prior to the 37th week of gestational age, was collected *via* medical chart review, which included gestational age of delivery. Secondary outcomes included PTB prior to the 34th, 32nd, and 28th weeks of gestational age.

Local gunshot data were collected from the Syracuse Police Department’s COMPSTAT reports,^32^ which collects week-by-week records of various crimes committed in the three districts of the City of Syracuse. Specifically, we focused on the number of gunshots fired in each of the districts. Prior research has shown that these types of crimes operate better under an area-specific model (*i.e.*, neighborhood) rather than an individual-specific model (*i.e.*, within 1 mile of an individual’s home).^33^ We separated gunshots into an acute-phase stressor and a chronic-phase stressor. The acute-phase stressor included the total number of gunshots in the person’s district the week prior to that person’s particular date of delivery. The chronic-phase stressor included the total number of gunshots in that district over the entire 2-year study period.

Comorbidities were also collected *via* chart abstraction. These included maternal age in years, self-identified maternal race/ethnicity (White, Black, Hispanic, Asian, other), insurance status (public or private), drug use (tobacco use or not, marijuana use or not, and other illicit drug use or not), body mass index (BMI; both the numerical value and the categorical split between BMI ≥35 kg/m^2^ or <35 kg/m^2^), pregestational hypertension or not, pregnancy-induced hypertension (gestational hypertension, preeclampsia, eclampsia, HELLP, *etc.*) or not, pregestational diabetes (type I or type II) or not, and gestational diabetes or not.

We used the three districts to divide the demographic and comorbid conditions. Distribution of demographic factors, comorbidities, and PTB was analyzed using means and standard deviations (SDs) for continuous variables and proportions for categorical variables. The distributions were compared across districts using analysis of variance for continuous variables, and Pearson’s chi-squared test for categorical variables. The frequency of acute-phase and chronic-phase gunshots and the frequency of PTB at various gestational age cutoffs were calculated for the study overall and for each district. We compared the average gunshots in those with and without PTB using Student’s *t*-test. Finally, we used unadjusted and adjusted logistic regressions. We used unadjusted models to associate acute-phase and chronic-phase gunshots with PTB. In the adjusted models, we included race, pregestational hypertension, pregnancy-induced hypertension, pregestational diabetes, gestational diabetes, insurance type, and all drug use (combining tobacco use, marijuana use, and illicit drug use). Two-sided *p*-values <0.05 were used to determine statistical significance. Those with missing data were removed from that specific analysis. Statistical analysis was completed *via* Stata version 16 (StataCorp, College Station, TX).

## Results

A total of 1675 unique deliveries were reviewed. These were split into living in the North district (*n* = 811, 48%), Southeast district (*n* = 401, 24%), and Southwest district (*n* = 463, 28%).

Demographic factors are shown in Table 1. Overall, the study population was on average 28.7 years old (SD = 6.3 years) and delivered an infant on average at 38 weeks and zero days of gestational age (SD = 2.7 weeks). Of these, 66% delivered vaginally. A majority was non-White (69%) and had public insurance (72%). Comorbid conditions included 38% obesity (BMI >35 kg/m^2^), 5% and 3% with pregestational hypertension and pregestational diabetes, respectively, and 7% for both pregnancy-induced hypertension and gestational diabetes.

**Table 1.** Demographic Characteristics of Study Sample^a^

	All (*n* = 1675)	North (*n* = 811)	Southeast (*n* = 401)	Southwest (*n* = 463)	*p*
Age (years)	28.7 (6.3)	29.1 (6.2)	29.3 (5.9)	27.6 (6.6)	<0.001
Parity	1.6 (1.9)	1.7 (2.0)	1.2 (1.6)	1.8 (1.7)	<0.001
Gestational age (weeks)	38 (2.7)	38 (2.59)	38 (2.6)	38 (2.9)	0.01
Vaginal delivery	1107 (66%)	542 (67%)	262 (65%)	303 (65%)	
Race^b^					
White	522 (31%)	299 (37%)	121 (30%)	102 (22%)	<0.001
Black	723 (43%)	287 (35%)	174 (43%)	262 (57%)	
Hispanic	177 (11%)	68 (8%)	40 (10%)	69 (15%)	
Asian	73 (4%)	47 (6%)	21 (5%)	5 (1%)	
Other	179 (11%)	110 (14%)	45 (12%)	24 (5%)	
Race					
White	522 (31%)	299 (37%)	121 (30%)	102 (22%)	<0.001
Non-White	1153 (69%)	512 (63%)	280 (70%)	361 (78%)	
Medicaid	1200 (72%)	611 (75%)	217 (54%)	372 (80%)	<0.001
Drugs					
Tobacco	208 (12%)	96 (12%)	36 (9%)	76 (16%)	0.003
Rec	181 (11%)	89 (11%)	28 (7%)	64 (14%)	0.005
Any^c^	322 (19%)	147 (18%)	56 (14%)	119 (26%)	<0.001
BMI (kg/m^2^)					
Avg	33.56 (8.0)	33.58 (8.3)	33.07 (7.7)	33.94 (7.96)	0.28
>35	639 (38%)	311 (38%)	150 (37%)	178 (38%)	0.94
Hypertension					
Pre	91 (5%)	32 (4%)	18 (5%)	41 (9%)	<0.001
Pregnancy^d,e^	122 (7%)	59 (7%)	29 (7%)	34 (7%)	1
Diabetes					
Pre^f^	45 (3%)	25 (3%)	6 (2%)	14 (3%)	0.24
Pregnancy^d^	109 (7%)	55 (7%)	21 (5%)	33 (7%)	0.48

^a^ All are mean (standard deviation) or *N* (%). *p*-values from ANOVA for continuous variables and Pearson’s chi-squared test for categorical variables.

^b^ One person from Southwest district did not report race.

^c^ Includes tobacco use, alcohol use, marijuana use, or any other illicit drug use.

^d^ In current pregnancy.

^e^ Includes gestational hypertension, preeclampsia, eclampsia, HELLP syndrome.

^f^ Includes type I or type II diabetes.

ANOVA, analysis of variance.

Demographics differed significantly between districts. The Southwest district had a significantly larger number of non-White people, public insurance users, and tobacco and recreational drug users than the other districts (all *p* < 0.05 for overall differences between districts). This district also had a larger number of preexisting hypertension (both *p* < 0.05). Finally, the Southwest district had a statistically significantly younger birthing population (both *p* < 0.05). Pregnancy-induced hypertension and all forms of diabetes mellitus did not differ between districts.

Aligning with the demographic differences, Table 2 shows the differences in gunshots and PTB between the districts. The Southwest district had a significantly higher number of mean gunshots in both the acute phase (Southwest 8.7 [SD = 5.6], North 3.3 [SD = 3.2], Southeast 1.1 [SD = 1.5], *p* < 0.001) and chronic phase (Southwest 285.5 [SD = 94.5], North 112.3 [SD = 50.2], Southeast 49.2 [SD = 25.3], *p* < 0.001) as compared with the North district, which had more than the Southeast district. The frequency of PTB when defined as birth prior to the 37th week of gestation differed significantly between districts (Southwest *n* = 82 [18%], North *n* = 106 [13%], Southeast *n* = 46 [12%], *p* = 0.018). However, there was no significant difference in the secondary outcomes of PTB defined at prior to 34, 32, or 28 weeks (all *p* > 0.05).

**Table 2.** Mean Number of Gunshots in Each District in the Acute Phase and Chronic Phase and Frequency of Preterm Birth or Preterm Premature Rupture of Membrane Based on District^a^

	All (*n* = 1675)	North (*n* = 811)	Southeast (*n* = 401)	Southwest (*n* = 463)	*p*
Gunshots in acute phase	4.3 (4.7)	3.3 (3.2)	1.1 (1.5)	8.7 (5.6)	<0.001
Gunshots in chronic phase	143.5 (111.0)	112.3 (50.2)	42.9 (25.3)	285.5 (94.5)	<0.001
PTB <37 weeks	234 (14%)	106 (13%)	46 (12%)	82 (18%)	0.018
PTB <34 weeks	81 (5%)	37 (5%)	15 (4%)	29 (6%)	0.2
PTB <32 weeks	50 (3%)	25 (3%)	10 (3%)	15 (3%)	0.79
PTB <28 weeks	23 (1%)	10 (1%)	5 (1%)	8 (2%)	0.74

^a^ All are mean (standard deviation) or *N* (%). *p*-values from ANOVA for continuous variables and Pearson’s chi-squared test for categorical variables.

PTB, preterm birth.

Table 3 directly compares the frequency of gunshots in those who did and did not meet the primary and secondary outcomes. Gunshots did not significantly differ in any of the prespecified outcomes, including PTB prior to 37, 34, 32, and 28 weeks (all *p* > 0.05).

**Table 3.** Mean Number of Gunshots in Acute Phase and Chronic Phase Overall and by District, Separated by Presence or Absence of Preterm Birth

	Mean gunshots (SD) (*n* = 1675)	*p* ^a^	North (*n* = 811)	Southeast (*n* = 401)	Southwest (*n* = 463)
Acute					
≥37 weeks	4.23 (4.76)	0.518	3.27 (3.23)	1.18 (1.6)	8.85 (5.7)
<37 weeks	4.44 (4.58)		3.39 (2.84)	0.61 (0.83)	7.96 (5.21)
≥34 weeks	4.29 (4.78)	0.195	3.3 (3.21)	1.13 (1.56)	8.88 (5.64)
<34 weeks	3.58 (3.78)		2.95 (2.4)	0.53 (0.92)	6 (4.63)
≥32 weeks	4.29 (4.77)	0.173	3.3 (3.2)	1.13 (1.55)	8.78 (5.65)
<32 weeks	3.36 (3.52)		2.76 (2.5)	0.5 (0.97)	6.27 (4.04)
≥28 weeks	4.27 (4.75)	0.537	3.28 (3.19)	1.12 (1.55)	8.74 (5.63)
<28 weeks	3.65 (3.56)		3.3 (2.11)	0.2 (0.45)	6.25 (4.2)
Chronic					
≥37 weeks	141.51 (109.59)	0.064	113.05 (50.13)	43.37 (25.66)	285.61 (94.46)
<37 weeks	155.97 (118.67)		106.92 (50.75)	38.8 (21.69)	285.11 (95.3)
≥34 weeks	143.66 (110.92)	0.835	113.29 (49.67)	42.7 (25.34)	287.59 (93.73)
<34 weeks	141.02 (112.91)		90.35 (57.05)	46.53 (23.26)	254.55 (102.2)
≥32 weeks	143.95 (110.94)	0.38	113.09 (49.8)	42.8 (25.36)	286.36 (94.06)
<32 weeks	129.96 (112.68)		85.56 (56.98)	44.9 (21.63)	260.67 (107.61)
≥28 weeks	143.49 (110.81)	0.914	112.44 (50.05)	42.93 (25.33)	285.68 (94.32)
<28 weeks	146 (125.37)		96.4 (63.71)	36.2 (18.94)	276.63 (111.12)

^a^ *p*-values from Student’s *t*-test comparing average gunshots in those with and without PTB.

Table 4 shows the odds ratios (ORs) for the association between gunshots and PTB, both unadjusted and adjusted. Gunshots, both unadjusted and when adjusted, were not associated with PTB at any gestational age measured. We did find that in both the acute phase and chronic phase, pregestational hypertension is associated with PTB prior to 37 weeks (acute OR = 3.381, confidence interval [CI]: 2.116–5.403; chronic OR = 3.328, CI: 2.081–5.322; both *p* < 0.01) and 34 weeks (acute OR = 2.225, CI: 1.038–4.770; chronic OR = 2.194, CI: 1.026–4.696; both *p* < 0.05), pregnancy-induced hypertension was associated with PTB prior to 32 weeks (acute OR = 2.492, CI: 1.122–5.534; chronic OR = 2.444, CI: 1.100–5.429; both *p* < 0.05), gestational diabetes was associated with PTB prior to 37 weeks (acute OR = 1.656, CI: 1.007–2.726; chronic OR = 1.656, CI: 1.007–2.725; both *p* < 0.05), and any drug use was associated with PTB prior to 32 weeks (acute OR = 2.015, CI: 1.074–3.779; chronic OR = 2.029, CI: 1.079–3.814; both *p* < 0.05). Chronic-phase gunshots trended to association with PTB prior to 37 weeks (chronic OR = 1.001, CI: 1.000–1.002, *p* = 0.05).

**Table 4.** Unadjusted and Adjusted Odds of Preterm Birth and Preterm Premature Rupture of Membrane Based on Confounding Comorbidities

	Acute				Chronic			
	<37 weeks	<34 weeks	<32 weeks	<28 weeks	<37 weeks	<34 weeks	<32 weeks	<28 weeks
Gunshot unadjusted OR (CI)	1.009	0.965	0.952	0.97	1.001	1	0.999	1
	(0.981–1.039)	(0.915–1.018)	(0.886–1.022)	(0.879–1.069)	(1.000–1.002)	(0.998–1.002)	(0.996–1.001)	(0.997–1.004)
Gunshot adjusted OR (CI)^a^	1.001	0.951	0.94	0.96	1.001	0.999	0.998	1
	(0.972–1.031)	(0.901–1.004)	(0.876–1.010)	(0.871–1.059)	(0.999–1.002)	(0.997–1.001)	(0.995–1.001)	(0.996–1.004)
Pre-HTN OR (CI)	3.381^*^	2.225**	1.478	1.352	3.328^*^	2.194**	1.482	1.322
	(2.116–5.403)	(1.038–4.770)	(0.497–4.399)	(0.286–6.388)	(2.081–5.322)	(1.026–4.696)	(0.499–4.402)	(0.282–6.206)
Preg-HTN OR (CI)	1.61	1.913	2.492**	0.586	1.615	1.879	2.444**	0.572
	(0.996–2.603)	(0.947–3.864)	(1.122–5.534)	(0.0771–4.454)	(0.999–2.611)	(0.930–3.794)	(1.100–5.429)	(0.0752–4.354)
Pre-DM OR (CI)	1.987	2.341	2.578	3.393	1.975	2.249	2.476	3.275
	(0.972–4.059)	(0.853–6.424)	(0.727–9.144)	(0.702–16.39)	(0.968–4.031)	(0.826–6.127)	(0.702–8.729)	(0.686–15.64)
Preg-DM OR (CI)	1.656**	0.517	0.576	N/A^b^	1.656**	0.525	0.583	N/A^b^
	(1.007–2.726)	(0.158–1.689)	(0.136–2.437)		(1.007–2.725)	(0.161–1.712)	(0.138–2.467)	
Medicaid OR (CI)	0.973	1.288	0.887	0.919	0.95	1.252	0.877	0.888
	(0.699–1.354)	(0.741–2.240)	(0.458–1.715)	(0.343–2.462)	(0.682–1.323)	(0.720–2.177)	(0.453–1.697)	(0.331–2.380)
All drug OR (CI)	1.316	1.451	2.015**	2.063	1.3	1.443	2.029**	2.055
	(0.936–1.850)	(0.861–2.445)	(1.074–3.779)	(0.815–5.220)	(0.924–1.828)	(0.855–2.435)	(1.079–3.814)	(0.810–5.212)

^a^ All adjusted models included race, pre- and preg-HTN, pre- and preg-DM, insurance, marital status, and all drug use.

^b^ None of the women who delivered at <28 weeks developed DM during pregnancy; therefore, an OR could not be calculated.

^*^ *p* < 0.01; ^**^p < 0.05.

CI, confidence interval; DM, diabetes mellitus; HTN, hypertension; NA, not available; OR, odds ratio; pre, pregestational; preg, pregnancy-induced; PPRM, preterm premature rupture of membrane.

## Discussion

Our study found that the three districts of the City of Syracuse, New York, had significant disparities in socioeconomic variables, gunshot frequencies, and PTB. However, the differences in gunshots were not associated with different frequencies in PTB, suggesting confounding factors were more strongly associated with PTB in this population. Pregestational hypertension, pregnancy-induced hypertension, gestational diabetes, and drug use were some of the socioeconomic factors found to be associated with the incidence of PTB.

This was the first study we are aware of that tried to link gunshot frequency with PTB. Our results differ from the findings of somewhat-related articles focusing on other forms of crime and negative pregnancy outcomes. The closest study was based in the City of Chicago, which looked at just over 34,000 births.^22^ Combining all crimes (including gunshots) committed in a person’s local neighborhood, they found small differences in PTB (as defined only as birth <37 weeks gestation) with increasing levels of overall crime. One study^33^ found a similar association with overall neighborhood crime (including gunshots) and PTB, but these findings are small and utilized a broader composite of crime. Another larger study out of North Carolina found no strong association between crime and PTB (OR = 1.5, CI: 0.9–2.6).^34^ A more recent study out of California found a slight increase in PTB by ∼0.5% in high-violence areas.^35^

Strengths of this study include the array of various socioeconomic zip-district zones and the wide range of different frequencies of gunshots. We have an ethnically and socioeconomically diverse population, which improves this study’s external validity. The Syracuse Police Department COMPSTAT dataset is a highly robust and detailed system using the ShotSpotter (SoundThinking Inc., Menlo Park, CA) gunshot detection system that boasts an average of 80% true positive detection rate and can generally triangulate each gunshot down to a specific address.^36,37^

The limitations of our study include an overall smaller number of participants. This may have limited the number of PTBs at the 34, 32, and 28 weeks of gestation cutoffs given the overall rarity of births at these gestational ages. This sample size limited our power to detect the small associations seen in the above prior research. For example, we would have needed ∼34,000 births to see the association found in the Chicago study.^22^ Our study had an 87% power to detect an OR of 1.5 but only a 38% power to detect an OR of 1.25.

We assigned each gunshot to a specific zip code-district area of the city but were unable to measure the exact distance of each gunshot to an individual’s home address. This may limit the effect each gunshot has on the perceived stress level and pregnancy outcome, as gunshots may have occurred either close by or far away (and altering the emotional impact that each single gunshot has) from each person’s home address while still counting as a gunshot. A few of the demographic factors were also patient reported (tobacco use, drug use, weight, and height for BMI), which are subject to inaccurate reporting or recording. The study took place during the active years of the COVID-19 pandemic, which may have impacted overall stress levels and the number of gunshots compared with prior nonpandemic years in other similar studies.^38^

This study also highlights the challenges that studies looking at PTB and looking at stress have. While it is likely that those living with more stressful lifestyles, whether it be emotional, physical, or social, are prone to a higher frequency of poor obstetrical outcomes, it can be quite challenging to delineate which specific aspects of stress contribute to the negative outcome. Stress, or allostatic load, is a collection of various lifestyle and personal factors that equate to physiological and psychological changes, and their effects on health outcomes can greatly differ based on how each individual perceives its impact. Likewise, PTB is a complex obstetric outcome that can be caused by a variety of factors, a few of which alone contribute greatly to an increased frequency. Rather, it is the complex overlay of many demographic and medical comorbid conditions that leads to the gestational age at which infants are born. These factors can make studies trying to delimitate specific risk factors practically difficult and with difficult integration into clinical practice.

## Conclusion

Overall, this study shows that the use of acute-phase and chronic-phase gunshots as a method to simulate stress levels is not associated with the development of PTB in this population.

## Abbreviations Used

BMI: body mass index
COMPSTAT: comparative statistics
PTB: preterm birth
